# Therapeutic potential of single-nucleotide polymorphism-mediated IL6R inhibitors in ankylosing spondylitis treatment

**DOI:** 10.3389/fmed.2024.1368346

**Published:** 2024-05-21

**Authors:** Ding-Qiang Chen, Wen-Bin Xu, Zhi-Qiang Que, Ke-Yi Xiao, Nai-Kun Sun, Di-Xin Cai, Jin-Yi Feng, Gang Rui

**Affiliations:** ^1^Department of Orthopedics, The First Affiliated Hospital of Xiamen University, Xiamen, China; ^2^The School of Clinical Medicine, Fujian Medical University, Fuzhou, China; ^3^Department of Orthopedics, The First Affiliated Hospital of Xiamen University, School of Medicine, Xiamen University, Xiamen, China

**Keywords:** IL6R, Mendelian randomization, causality, drug target, ankylosing spondylitis

## Abstract

**Objective:**

Interleukin-6 (IL-6) is a multiple-effect cell factor implicated in the etiopathogenesis of several rheumatologic disorders. The blockade of the IL-6 pathway via IL6R inhibitors effectively treats these disorders. However, the clinical significance of the IL6R blockade for ankylosing spondylitis (AS) therapy remains controversial. With advances in genomics, increasing evidence has revealed the role of heritability in the etiology of disease, and Mendelian randomization (MR) analyses are being used more broadly to infer causation. Therefore, this MR study aims to evaluate the potential therapeutic utility of IL6R-targeted approaches in AS.

**Methods:**

The C-reactive protein (CRP) level was used as an exposure factor, and rheumatoid arthritis (RA) was used as a positive control. As-related genome-wide association study (GWAS) data were used as the primary outcome of drug-targeted MR analyses to test the relation between IL6R blockers and AS. Inverse variance weighting (IVW) is the primary analytical approach. Various sensitivity tests were performed to check the robustness and trustworthiness of the causality estimation, including consistency, heterogeneity, and pleiotropy analyses. In addition, repeated analysis was conducted using different GWAS data related to exposures and outcomes to examine the results for stability.

**Results:**

According to the IVW results, IL6R inhibitors significantly reduced the risk of AS in ukb-b-18194 (OR: 0.995, 95% CI 0.993–0.996, *P* = 5.12 × 10^−08^) and ukb-a-88 (OR: 0.994, 95% CI 0.993–0.996, *P* = 6.25 × 10^−15^). Moreover, repeated analyses were performed using different exposure-related GWAS data, yielding similar results, ukb-b-18194 (OR: 0.995, 95% CI 0.993–0.997, *P* = 1.25 × 10^−06^) and ukb-a-88 (OR: 0.995, 95% CI 0.994–0.997, *P* = 7.81 × 10^−09^). Heterogeneity analyses and pleiotropy analyses indicated no significant heterogeneity or pleiotropy.

**Conclusion:**

This MR analysis result further validates that the IL-6 pathway may contribute to the pathogenesis of AS and that the inhibition of IL6R reduces the risk of AS. These findings may guide future studies and provide more favorable drug treatment options for people at high risk of AS.

## Introduction

Ankylosing spondylitis (AS) is a long-term progressing inflammatory disorder mainly involving the medial skeleton, where the synovial tissue, spine ligaments, vertebral disk, and small joints become aseptically inflamed (1). The clinical features are back and sacroiliac joint pain and rigidity, usually accompanied by peripheral joint inflammation, attachment point inflammation, and acute anterior uveitis (2).

AS is characterized by long-term inflammation and a high disability rate, and its current main coping strategies include health education, physical therapy, medication, and surgical intervention. Non-steroidal anti-inflammatory drugs (NSAIDs) are the first-line therapeutic drugs for AS, primarily utilized for improving the patient's symptoms (3). In addition, targeted drugs such as anti-IL-17 and TNF-α are also frequently used to treat AS (4). Surgery may be used for some patients with AS for whom non-surgical treatment is ineffective (5, 6). However, long-term drug treatment has significant toxic side effects, surgical treatment is expensive, and the prognosis is relatively general, which severely affects the patient's psychological and physical health and puts tremendous pressure on the individuals and society. In addition, the etiology of AS is highly complex. Genetic, immunologic, microbiologic, and endocrinologic factors are generally believed to be involved in the onset and development of AS, with hereditary factors being the leading cause (7, 8). Nevertheless, the exact pathophysiologic process of AS is yet incompletely recognized. Hence, there is a need for more research regarding its pathogenic factors.

IL-6 is a polymorphic-promoting inflammatory cellular factor proven for its critical function in immune dysfunction, initiation, and persistence of inflammatory reactions within diverse autoimmune joint inflammations and other inflammation disorders of the gastrointestinal, cardiac, and optical systems (9). A meta-analysis of 13 case–control trials, including 514 individuals with AS and 358 controls, demonstrated that elevated serum IL-6 levels are markedly related to the development of AS (10). In addition, Du et al. confirmed that AS patients with sacroiliitis and other arthritis conditions (e.g., knee and neck) have higher serum IL-6 levels and that IL-6 levels are significantly related to CT image classifications of sacroiliitis through case–control studies, suggesting that IL-6 has a significant effect on the progress of bony impairment in AS patients (11). Inhibition of the IL-6 pathway via anti-IL6R showed promising results in juvenile idiopathic joint inflammation, adult-type Still's disease, macrovascular vasculitis, systematic sclerosis, and rheumatic polymyalgia. However, the effect of anti-IL6R in AS remains controversial, and the clinical significance of the IL-6 blockade has yet to be observed (12, 13).

Drug Mendelian randomization (MR) is a novel research method used in drug discovery and effect prediction. This method is based on the downstream products (biomarkers) of the target proteins, single-nucleotide polymorphisms (SNPs) near the genes encoding the target proteins that have a significant effect on the biomarkers as an instrumental variable (IV), the concentration of the biomarkers that serve as the exposures, and the disorders that serve as the outcomes, and the impact of the protein target on the disease under study is verified by MR (14). MR has been broadly employed to examine the underlying causality between exposures and outcomes due to its better credibility than conventional observational research in deducing causality, conquering confounding factors, measuring errors, and reversing causality (15). This study collected and analyzed relevant GWAS pooled data by drug-targeted MR to examine the association between IL6R inhibitors and AS.

## Materials and methods

### Study design

**Figure 1:**
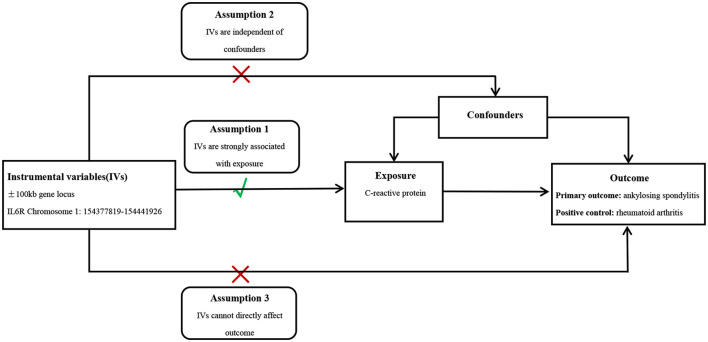
Conceptual diagram of MR study design.

It is well-known that IL6R inhibitors can be used to treat rheumatoid arthritis (RA); therefore, pooled GWAS data of RA provided a positive control for testing the reliability of inverse variance (IV). Genetic instruments in MR should satisfy three hypotheses: SNPs are linked to exposure, SNPs are not linked to confounding elements that may intervene in the causality between exposures and outcomes, and SNPs correlate with the outcomes only through exposures (Figure 1).

### Selection of instrumental variables

Priority was given to the GWAS databases with more samples and SNPs. GWAS pooled data of CRP were downloaded from the European Bioinformatics Institute (EBI) database, containing 353,466 individuals of European origin. Instrumental Variables (IVs) targeting IL6R were obtained by identifying SNPs in the ±100-kb range of the IL6R locus and are associated with CRP serum levels (association *P-*value < 5 × 10^−8^), i.e., they could mimic the effects of IL6R inhibitors since CRP serum levels are a reliable downstream biomarker of IL-6 signaling (16). To ensure that a sufficient number of SNPs representative of the traits analyzed in this drug-targeted MR analysis were included and to minimize the effect of strong linkage disequilibrium (LD) to a certain extent, we finally fixed the critical value of LD (*r*^2^ < 0.3) by referring to the literature wherein excellent results have been achieved in the past (17–19). In addition, to prevent weak instrumental bias, only SNPs with *F*-statistic >10 were included in the analysis. Finally, significant SNPs that met the criteria were retained (Supplementary Tables S1, S2). Additionally, for the stability of the results, exposure-related pooled data from other GWAS data (containing 436,939 people of European descent) were used for replicate analyses. AS served as the major result for MR analysis, whereas the RA was chosen as a positive control. These datasets were all derived from European populations. The RA data contain 5,201 cases and 457,732 controls. In addition, pooled data of AS from two different GWAS studies were collected for analysis (Table 1).

**Table 1:** Summary of GWAS datasets included in this study.

**Phenotype**	**Sample size (case/control)**	**Number of SNPs**	**Population**	**Units**
**Exposure**				
C-reactive protein levels (ebi-a-GCST90018950)	353,466	19,057,467	European	Not applicable
C-reactive protein levels (ebi-a-GCST90025959)	436,939	4,231,728	European	Not applicable
**Outcomes**				
Rheumatoid arthritis (ukb-b-9125)	462,933 (5,201/457,732)	9,851,867	European	SD
Ankylosing spondylitis (ukb-b-18194)	462,933 (1,296/461,637)	9,851,867	European	SD
Ankylosing spondylitis (ukb-a-88)	337,159 (968/336,191)	10,894,596	European	SD

SNP, single nucleotide polymorphism.

### Data analysis

The inverse variance weighting (IVW) was adopted as the predominant analytical choice. Moreover, the MR Egger, weighted median, simple mode, and weighted mode approaches were used for validation (20). The IVW methods can stably predict the causation between exposures and outcomes under the hypothesis that IV assumptions are met for each genetic variable (21). Under weaker assumptions, the Egger and weighted median approaches can estimate stable causality for several heritable variants from aggregated data. The weighted median estimation produces concordant causality estimation, even if as much as half of the information is derived from the null IV hypothesis (22). In addition, the MR-Egger regression and MR-pleiotropy residual sum outlier (MR-PRESSO) methods were used in identifying and adjusting for pleiotropy. The MR-Egger regression analyses test for and account for imbalanced pleiotropy by combining pooled data estimation of the causality of several single variables (23). MR-Egger uses a weighted linear regression of genetic outcome data on genetic exposure data. The slope of the linear regression represents causality estimation, and the average level of pleiotropy of genetic variants was expressed as the intercept (24). Cochran's *Q* statistics were used to evaluate the heterogeneity among SNP estimations (25). A *P*-value of > 0.05 indicates no heterogeneity and horizontal pleiotropy. Moreover, to exclude confounding factors that may have affected the results of this analysis, traits directly associated with SNPs were searched through the online site PhenoScanner (26), and relevant data were included in the Supplementary Tables S1, S2 to ensure that all SNPs met the experimental hypotheses. Finally, the “leave-one-out” sensitivity test was carried out to gauge the robustness of the results through the removal of SNPs one by one. This analysis was performed using R 4.3.1. The “forestplot” package was employed to map the forest diagram, and the “MRPRESSO” package was applied for outlier detection.

## Results

### Positive control analyses

**Figure 2:**
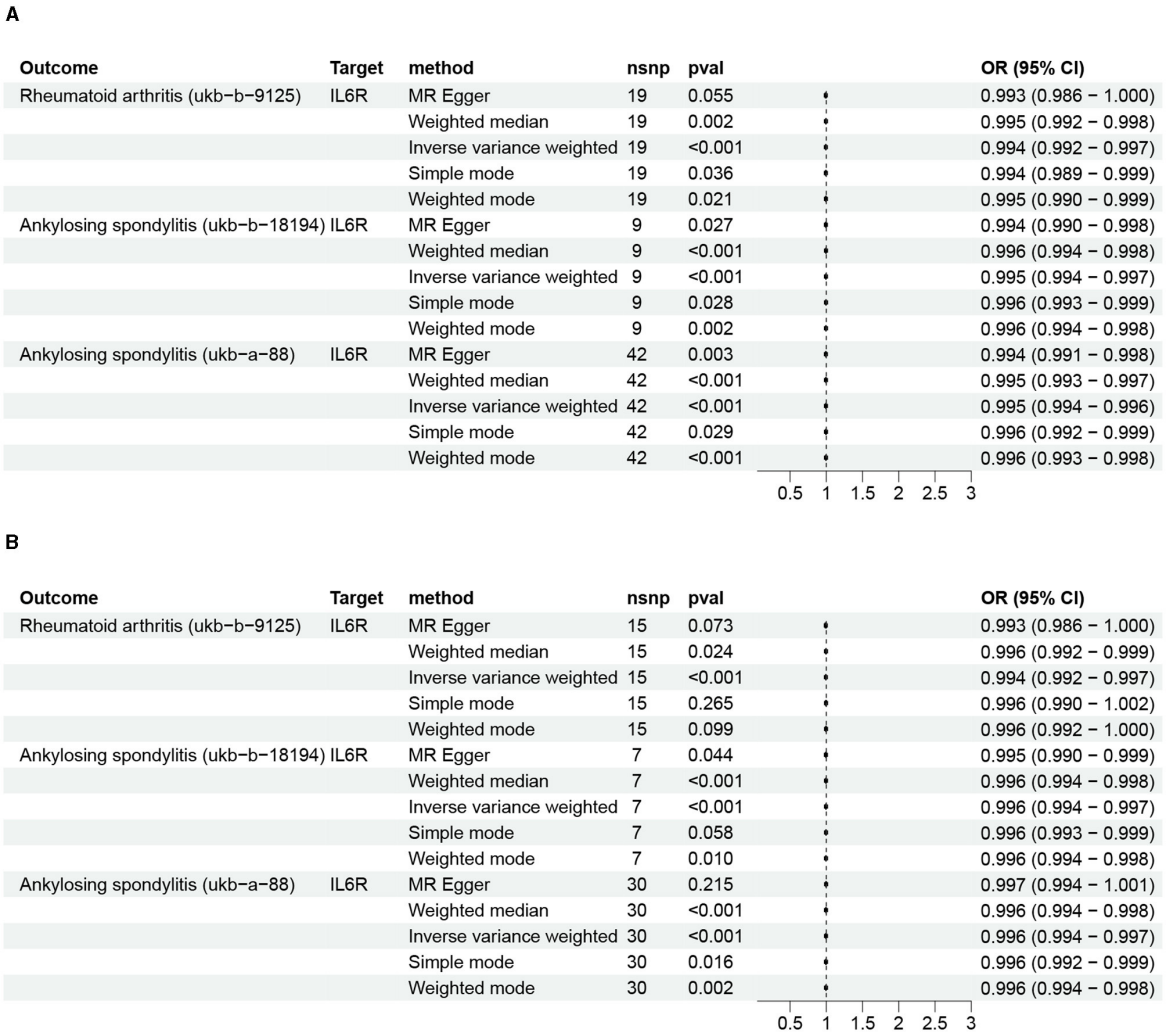
The impact of IL6R inhibitors on rheumatoid arthritis and ankylosing spondylitis. **(A)** Initial analysis; **(B)** repeated analysis.

The IVW results suggested that inhibition of IL6R can reduce the risk of RA (OR: 0.994, 95% CI: 0.991–0.996, *P* = 3.12 × 10^−6^). Moreover, the other four MR analysis approaches also generated similar results (Figure 2 and Supplementary Table S3). Repeated analysis with another exposure-related GWAS dataset also indicated that IL6R inhibitors could have a positive effect on the treatment of RA (OR: 0.994, 95% CI: 0.991–0.997, *P* = 3.47 × 10^−5^; Figure 2 and Supplementary Table S4). Various clinical trials have demonstrated the superior efficacy of the IL6R monoclonal antibody, which is currently authorized for treating RA and juvenile idiopathic arthritis (27). The reliability of the results of this analysis is well illustrated using RA as a positive control for the drug-targeted MR analysis.

### The causality between inhibitors of IL6R and AS

Analysis of data from two AS GWASs suggests that IL6R inhibitors can reduce the risk of AS: ukb-b-18194 (IVW: OR: 0.995, 95% CI 0.993–0.996, *P* = 5.12 × 10^−08^; MR Egger: OR: 0.994, 95% CI 0.990–0.998, *P* = 0.02; weighted median: OR: 0.995, 95% CI 0.993–0.997, *P* = 2.94 × 10^−05^; simple mode: OR: 0.996, 95% CI 0.993–0.998, *P* = 0.02; weighted mode: OR: 0.995, 95% CI 0.993–0.997, *P* = 2.43 × 10^−03^) and ukb-a-88 (IVW: OR: 0.994, 95% CI 0.993–0.996, *P* = 6.25 × 10^−15^; MR Egger: OR: 0.994, 95% CI 0.990–0.997, *P* = 2.91 × 10^−3^; weighted median: OR: 0.995, 95% CI 0.993–0.997, *P* = 1.40 × 10^−06^; simple mode: OR: 0.995, 95% CI 0.992–0.999, *P* = 0.02; weighted mode: OR: 0.995, 95% CI 0.993–0.998, *P* = 9.36 × 10^−04^; Figure 2 and Supplementary Table S3). Moreover, repeated analyses using data from different exposure-related genomic studies yielded similar results: ukb-b-18194 (IVW: OR: 0.995, 95% CI 0.993–0.997, *P* = 1.25 × 10^−06^) and ukb-a-88 (IVW: OR: 0.995, 95% CI 0.994–0.997, *P* = 7.81 × 10^−09^; Figure 2 and Supplementary Table S4).

### Sensitivity analysis

The heterogeneity and horizontal pleiotropy were assessed via application of Cochrane's *Q* and MR Egger regression equations (Tables 2, 3). No heterogeneity or horizontal pleiotropy was found when examining the causality between IL6R inhibitors and AS (Table 2).

**Table 2:** The result of heterogeneity and horizontal pleiotropic test.

**Outcomes**	**Drug target**	**Heterogeneity test**				**Horizontal pleiotropic test**		
		**Method**	**Q**	**Q_df**	**Q_Pval**	**Egger _intercept**	**SE**	***P*** **-value**
Rheumatoid arthritis (ukb-b-9125)	IL6R	MR Egger	9.213403873	17	0.933327889	−4.92E−05	0.000158	0.759195498
		Inverse variance weighted	9.310446691	18	0.95211147			
Ankylosing spondylitis (ukb-b-18194)	IL6R	MR Egger	8.208726772	7	0.314549065	−5.61E−05	9.70E−05	0.581481724
		Inverse variance weighted	8.600268161	8	0.377129821			
Ankylosing spondylitis (ukb-a-88)	IL6R	MR Egger	36.82548558	40	0.613956126	−2.31E−05	8.65E−05	0.790813381
		Inverse variance weighted	36.89679383	41	0.653524075			

IL6R, interleukin 6 receptor; SE, standard error.

**Table 3:** The result of heterogeneity and horizontal pleiotropic test for repeated analysis.

**Outcomes**	**Drug target**	**Heterogeneity test**				**Horizontal pleiotropic test**		
		**Method**	**Q**	**Q_df**	**Q_Pval**	**Egger _intercept**	**SE**	***P*** **-value**
Rheumatoid arthritis (ukb–b−9125)	IL6R	MR Egger	9.285963462	13	0.751019329	−7.59593E−05	0.00018281	0.684549226
		Inverse variance weighted	9.458607328	14	0.800598844			
Ankylosing spondylitis (ukb–b−18194)	IL6R	MR Egger	5.358814179	5	0.373675834	−6.67345E−05	0.00010238	0.543280409
		Inverse variance weighted	5.814189466	6	0.444323481			
Ankylosing spondylitis (ukb–a−88)	IL6R	MR Egger	19.54269175	28	0.880558077	9.54969E−05	0.00010197	0.357007804
		Inverse variance weighted	20.41978245	29	0.879400199			

IL6R, interleukin 6 receptor; SE, standard error.

To be more certain of the findings' stability, we similarly performed sensitivity analyses of our procedures using different exposure-related GWAS data, and encouragingly, the results still showed that our analysis did not exhibit significant heterogeneity or horizontal pleiotropy (Table 3). Leave-one-out analyses indicated that, after excluding any of the SNPs for RA and AS, the results did not show substantial differences (Supplementary Figure S1).

## Discussion

In this drug-targeted MR analysis, we selected 44 SNPs from the vicinity of IL6R to mimic the inhibitory effect of IL6R. RA was chosen as positive control to analyze the influence of SNPs on AS. The analysis results suggested that the risk of AS could be reduced by IL6R in both outcome datasets, aligning with previously published studies. To further validate the stability of analysis results, another exposure dataset containing 436,939 participants was chosen for a repeat analysis, which produced similar results.

IL-6, recognized initially as B-cell stimulating factor 2, is a typical cell factor with substantial bioefficacy on immunocytes like B-cells, T-cells, liver cells, hematopoietic cells, and intravascular endothelial cells (28). In addition, dysregulation of IL-6 and the production of its receptor has been connected to the etiopathogenesis of several disorders, such as multiple myeloma, self-immune disorders, and carcinoma of the prostate. Several studies have demonstrated the potential function of IL-6 in the etiopathogenesis of AS. François et al. found the presence of IL-6 in all tissues from four-fifths of AS patients by immune-histological inspection of open sacroiliac joint biopsy of AS patients and because IL-6 was more frequently pooled in advanced cases of AS compared to early cases (29). Many studies have also confirmed elevated serum IL-6 levels, and their relevance to acute-stage reactants, AS disease activity levels, and acute inflammatory changes were observed on imaging (30–34). Even though there is substantial proof that the IL-6 pathway has a crucial effect on the etiopathogenesis of AS, studies aimed at evaluating the clinical impact of the IL-6 pathway blockade in AS are scarce.

There is currently a very controversial debate about the effect of IL6R inhibitors in AS therapy. A systematic literature study spanning 8 years and integrating multiple databases to explore the evidence for a positive role of IL-6 inhibition in a variety of inflammatory diseases found no therapeutic effect of IL-6 inhibition in ankylosing spondylitis, psoriatic arthritis, and certain connective tissue diseases (35). However, in one case of AS in a 30-year-old man whose physical examination revealed severely decreased spine and hip mobility and a significant increase in markers of the acute-phase response, the patient's physical condition improved rapidly and clinical symptoms gradually improved during the 11 months of treatment with IL6R antagonists. On review, the patient was in good physical condition with no joint tenderness or swelling, morning stiffness lasting no more than 1 h, and inflammation indicators in the normal range (36). In addition, in another 34-year-old Japanese male AS patient treated with IL6R antagonists, the patient's Bath Ankylosing Spondylitis Disease Activity Index (BASDAI) (37) and Bath Ankylosing Spondylitis Functional Index (BASFI) (38) decreased gradually from 3.3 to 0.4 and from 2.7 to 0.1, respectively. The patient's CRP level decreased rapidly and remained at 0.04 mg/dL or lower via treatment. After 6 months, active knee inflammation and bone marrow edema also disappeared from the review images. In addition, leukocytosis, mild anemia, and low serum albumin levels observed before treatment were significantly improved, and no adverse events occurred throughout the treatment period (39).

AS is a long-term inflammatory disorder that is conventionally believed to be mediated by T lymphocytes (40). IL-6 influences many kinds of cells via its specialized receptor system and possesses a wide range of bioactivities. Its increased secretion is part of the pathogenesis of a variety of self-immune and inflammatory disorders (41). Among these, IL-6 is engaged in helper T-cell division and differentiation. When mitogens stimulate T cells, IL-6 significantly enhances T-cell proliferation and facilitates the discharge of various immunological factors (42, 43). Helper T cells (Th1 cells) are a subpopulation of CD4+ T cells that can release cellular factors like IFN-γ, IL-2, and TNF-α for activating other immunocytes and promoting cellular immunological reactions (44). A study on AS immunobiology by Yang and his team found that CCR4 expression on circulating CD4 T cells was significantly increased and correlated with the BASDAI and that overexpression of CCR4 as a chemokine receptor on CD4+ T cells, a Th2 cell chemokine receptor, demonstrated a significantly enhanced Th2 response in AS (45). Moreover, Wang et al. found that Th1/Th2 ratios were increased significantly in the mild and severe AS groups when compared with normal controls by obtaining pericytes from lesions and sera of 55 patients with AS and that imbalance of these T-cell subpopulations may lead to enhanced IFN-γ and thus promote a more severe inflammatory response in AS patients (46). Therefore, there may be a logical correlation between the role of IL-6 in mediating the development of AS, as IL-6 binds to its specialized receptor, IL6R, and produces biological activity that promotes the division and differentiation of helper T-cells and contributes to the progression of the inflammatory response in AS by influencing the activity of Th1 and Th2 and also their ratio. Furthermore, results from mouse-based immunological research suggest that co-stimulatory effects of IL-6 and TGF-β are critical for the differentiation of Th17 cells out of naive CD4+ T cells, the primary function of which is to protect against disease-causing bacteria like *Staphylococci, Mycobacteria, Klebsiella*, or fungal pathogens, yet excessive production of IL-17 by activated T cells can lead to autoimmune disorders. Unfettered IL-7 generation leads to the excretion of inflammatory agents by macrophages, endothelial cells, dendritic cells, and fibroblasts. Emission of these factors causes inflammation and joint damage in RA and AS (47–49). Therefore, blockade of the IL-6 pathway by IL6R antagonists should lead to positive results in AS treatment.

## Study strengths and limitations

There are two advantages to this analysis. First, it is the first study on the association between IL6R inhibitors and AS through large-scale GWAS data with the exclusion of confounders and reverse causality, which is very helpful in completely understanding the therapeutic potential of IL6R inhibitors in AS. Second, exposure and outcome data were obtained from different datasets through the two-sample study design, making the exposure and outcome data non-overlapping and minimizing bias. Nonetheless, the study also suffers from some unavoidable limitations. First, the data used in the study came mainly from online public databases, and due to differences in platforms and regions, some of the data may be heterogeneous. Second, the data used for AS were limited to the European population only. Due to the variability of populations in different regions, there may be some variability in the conclusions of this experiment when applied to other non-European populations. Third, this study focused on analyzing causality from a genetic perspective, and the exact underlying mechanisms require further analysis by essential trials. Finally, the effect values of the results of this analysis were not highly significant. The odds ratio (OR) value is a measure used in statistics to indicate whether two dichotomous variables are correlated or not and expresses the odds ratio of events happening, i.e., the probability of one set of events occurring to the probability of another set of events occurring. When the OR is close to 1, the exposure and control groups had similar odds of having an event (50). Although the OR of the results of this MR analysis is close to 1, it does not mean that the results of this study are not significant. First, the confidence intervals (CIs) for the results of this study were narrow, and all CIs were < 1, which suggests that the estimates are exact and that IL6R antagonists did reduce the risk of AS. Second, the data used in this study were all GWAS data from large samples, so the study results have a very high confidence level. Most importantly, AS has a significant global incidence, with the estimated incidence and prevalence ranging from 0.05 to 1.4/10,000 person-years and from 0.1 to 1.4%, respectively. AS typically affects patients in their 20s and tends to be observed more commonly in men. It poses serious physical and psychological health morbidity, which in turn affects work productivity and incurs substantial personal and social costs (51). Therefore, even minimal effects may have important implications for population health and support the clinical response to AS.

## Conclusion

In summary, this research provides genetic evidence that the proinflammatory cytokine receptor antagonist class of drug therapies, explicitly acting through the IL6R pathway, have a potentially causal effect in lowering AS risk. This finding informs future pharmacologic treatment choices for AS patients. It may reduce the incidence of AS and increase the patient's general prognosis and overall quality of life. Although some clinical evidence supports our results, more detailed studies on the role of IL6R antagonists are needed. Further genetic research may contribute to dissecting the effects of IL-6 signaling in AS. In addition, our research emphasizes the benefits of using genetic data to study drug repurposing and reduce the risk of AS.

## Data availability statement

The original contributions presented in the study are included in the article/Supplementary material, further inquiries can be directed to the corresponding authors.

## Ethics statement

Ethical approval was not required for the study involving humans in accordance with the local legislation and institutional requirements. Written informed consent to participate in this study was not required from the participants or the participants' legal guardians/next of kin in accordance with the national legislation and the institutional requirements.

## Author contributions

D-QC: Conceptualization, Methodology, Writing – original draft. W-BX: Writing – review & editing. Z-QQ: Validation, Writing – original draft. K-YX: Formal analysis, Writing – original draft. N-KS: Visualization, Writing – original draft. D-XC: Data curation, Software, Writing – original draft. J-YF: Formal analysis, Writing – review & editing. GR: Funding acquisition, Supervision, Writing – review & editing.
