# Supplementary material for: Therapeutic potential of single-nucleotide polymorphism-mediated IL6R inhibitors in ankylosing spondylitis treatment
Source: Front Med (Lausanne). 2024 May 21;11:1368346. doi: 10.3389/fmed.2024.1368346 (PMC11148286; doi:10.3389/fmed.2024.1368346)
Supplement: Supplementary file 1 [file Image_1.pdf]

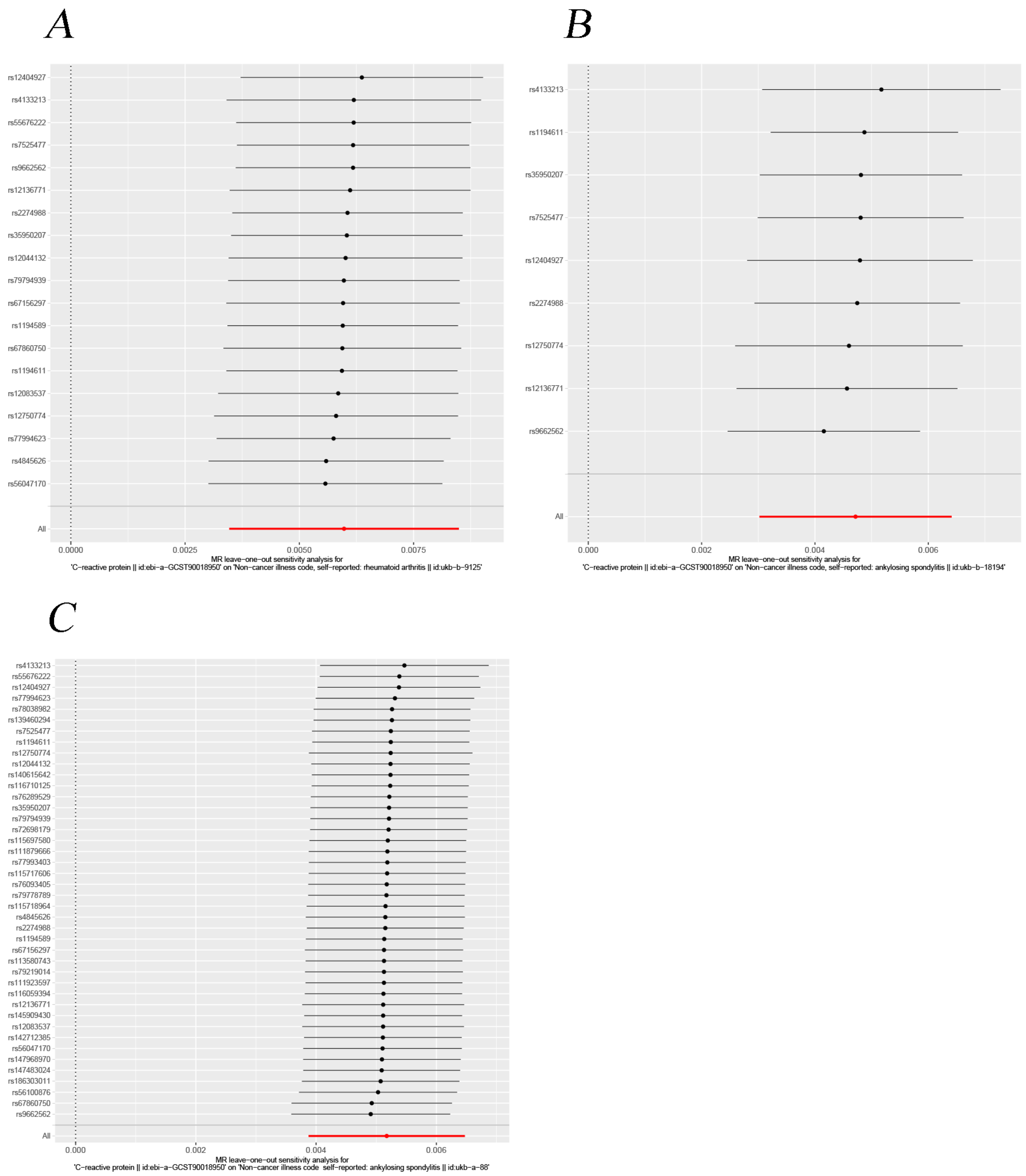

Fig. S1 Leave-one-out analysis analysis of IL6R on rheumatoid arthritis and ankylosing spondylitis. The leave-one-out method is used to evaluate the excessive impact of a single SNP on MR analysis if the comprehensive effect of the remaining SNPs is consistent with the main effect after removing one SNP. (A) Rheumatoid arthritis; (B) Ankylosing spondylitis (ukb-b-18194); (C) Ankylosing spondylitis (ukb-a-88). SNP, single nucleotide polymorphisms; IL6R, Interleukin 6 receptor.

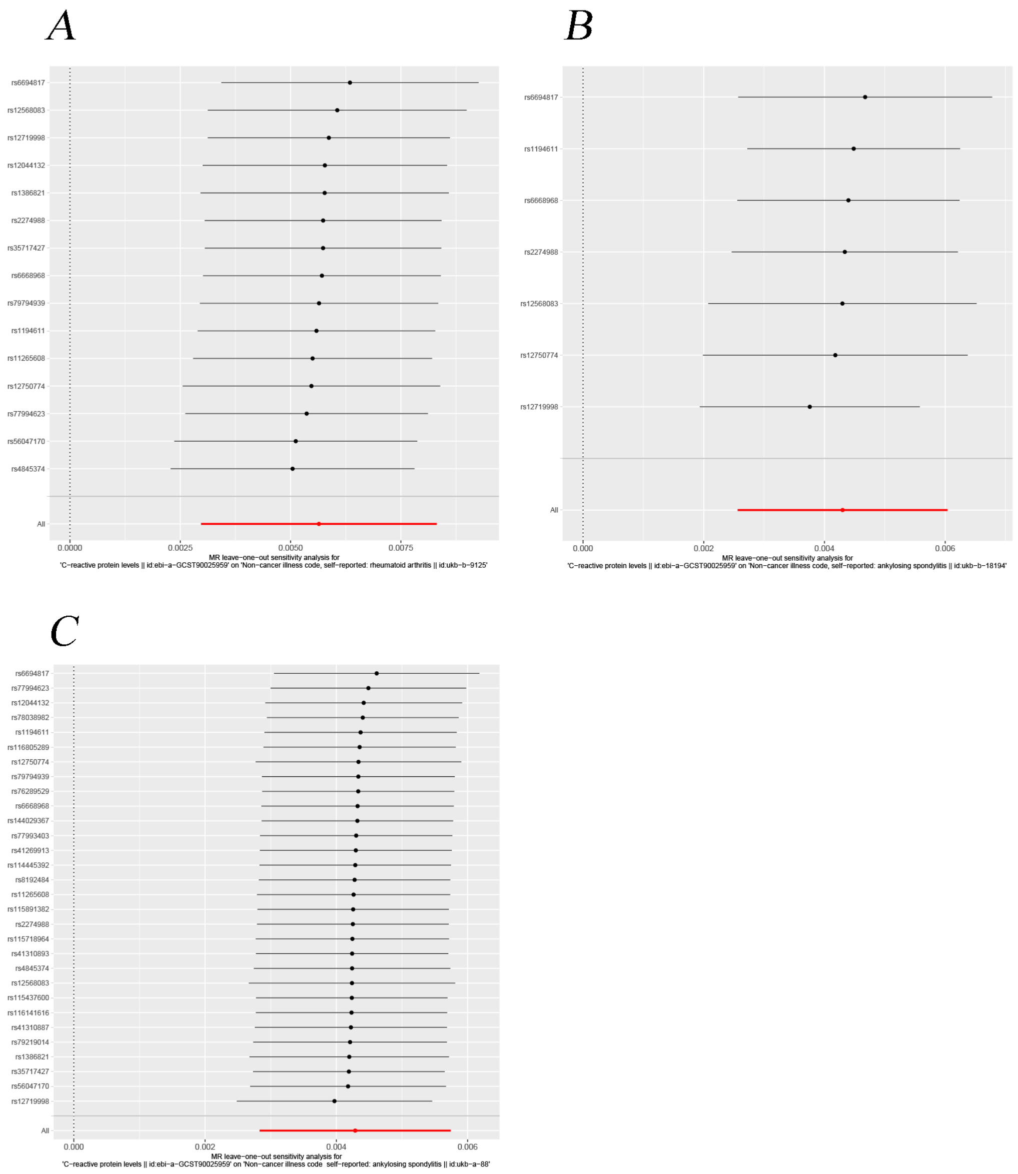

(A) Leave-one-out analysis analysis of IL6R on rheumatoid arthritis and ankylosing spondylitis in the repeated analysis. The leave-one-out method is used to evaluate the excessive impact of a single SNP on MR analysis if the comprehensive effect of the remaining SNPs is consistent with the main effect after removing one SNP. (A) Rheumatoid arthritis; (B) Ankylosing spondylitis (ukb-b-18194); (C) Ankylosing spondylitis (ukb-a-88). SNP, single nucleotide polymorphisms; IL6R, Interleukin 6 receptor.
